# Supplementary material for: Unique developmental trajectories of risk behaviors in adolescence and associated outcomes in young adulthood
Source: PLoS One. 2019 Nov 13;14(11):e0225088. doi: 10.1371/journal.pone.0225088 (PMC6853606; doi:10.1371/journal.pone.0225088)
Supplement: S1 Table — (DOCX) [file pone.0225088.s001.docx]

S1 Table. Confirmatory Factor Analysis (CFA) for each wave separately.

| **Factor loadings** | | | | | |
| --- | --- | --- | --- | --- | --- |
|  | **Wave 2** | **Wave 3** | **Wave 4** | **Wave 5** |  |
| Alcohol | .635 | 0.613 | .403 | .328 |  |
| Cannabis | .332 | .455 | .499 | .496 |  |
| Smoke | .606 | .594 | .625 | .497 |  |
| Externalizing | .472 | .597 | .413 | .361 |  |
|  |  |  |  |  |  |
| CFI | .978 | .999 | 1.000 | .989/.022 |  |
| AIC | 27641 | 29574 | 31133 | 28860 |  |
| BIC | 27710 | 29639 | 31199 | 2886 |  |
