# Supplementary material for: Unique developmental trajectories of risk behaviors in adolescence and associated outcomes in young adulthood
Source: PLoS One. 2019 Nov 13;14(11):e0225088. doi: 10.1371/journal.pone.0225088 (PMC6853606; doi:10.1371/journal.pone.0225088)
Supplement: S2 Table — a: model results for 14 and 16 years b: model results for 16 and 19 years c: model results for wave 14 to 19 years d: model results for 14 to 22 years. (DOCX) [file pone.0225088.s002.docx]

S2 Table. Measurement invariance (MI): Partial MI; factor loadings constraint to be equal

|  | **Wave 2^a^** | **Wave 3^b^** | **Wave 4^c^** | **Wave 5^d^** |  |
| --- | --- | --- | --- | --- | --- |
| Alcohol | .622 (.054) | .170 (.086) | .183 (.078) | .123 (.061) |  |
| Cannabis | .449 (.073) | .528 (.040) | .453 (.045) | .389 (.045) |  |
| Smoke | .647 (.068) | .660 (.057) | .650 (.067) | .496 (.060) |  |
| Externalizing | .294 (.077) | .480 (.050) | .481 (.036) | .468 (.040) |  |
|  |  |  |  |  |  |
| CFI | .786/.076 | .986/.026 | .842 | .890/.047 |  |
| AIC | 56728 | 59059 | 86301 |  |  |
| BIC | 56881 | 59219 | 86562 |  |  |

^a^: model results for 14 and 16 years ^b^: model results for 16 and 19 years ^c^: model results for wave 14 to 19 years ^d^: model results for 14 to 22 years
