# Supplementary material for: Unique developmental trajectories of risk behaviors in adolescence and associated outcomes in young adulthood
Source: PLoS One. 2019 Nov 13;14(11):e0225088. doi: 10.1371/journal.pone.0225088 (PMC6853606; doi:10.1371/journal.pone.0225088)
Supplement: S3 Table — a: model results for 14 and 16 years b: model results for 16 and 19 years c: model results for wave 14 to 19 years d: model results for 14 to 22 years. (DOCX) [file pone.0225088.s003.docx]

S3 Table. Measurement Invariance: Full MI; factor loadings and intercepts constrained to be equal.

|  | Wave 2^a^ | Wave 3^b^ | Wave 4^c^ | Wave 5^d^ |  |
| --- | --- | --- | --- | --- | --- |
| Alcohol | .456 (.187) | .594 (.092) | .546 (.082) | .476 (.073) |  |
| Cannabis | .611 (.112) | .514 (.049) | .489 (.050) | .4555 (.043) |  |
| Smoke | .273 (.102) | .747 (.060) | .757 (.066) | .692 (.072) |  |
| Externalizing | .049 (.017) | .247 (.052) | .290 (.048) | .283 (.039) |  |
|  |  |  |  |  |  |
| CFI/RMSEA | .209/.131 | .658/.107 | .147/.128 | .468/.096 |  |
| AIC | 57471 | 59801 | 88024 | 112591 |  |
| BIC | 57602 | 59923 | 88240 | 112919 |  |

^a^: model results for 14 and 16 years ^b^: model results for 16 and 19 years ^c^: model results for wave 14 to 19 years ^d^: model results for 14 to 22 years
