# Supplementary material for: Unique developmental trajectories of risk behaviors in adolescence and associated outcomes in young adulthood
Source: PLoS One. 2019 Nov 13;14(11):e0225088. doi: 10.1371/journal.pone.0225088 (PMC6853606; doi:10.1371/journal.pone.0225088)
Supplement: S4 Table — (DOCX) [file pone.0225088.s004.docx]

S4 Table. Sex differences with partial MI and without alcohol (only factor loadings constrained)

|  | Wave 2  Male/female | Wave 3  Male/female | Wave 4  Male/female | Wave 5  Male/female | Model fit when constrained to be equal |
| --- | --- | --- | --- | --- | --- |
| Cannabis | .618/.898 | .426/.735 | .195/.681 | .213/.571 |  |
| Smoke | .433/.272 | .558/.518 | .504/.458 | .490/.375 |  |
| Externalizing | .222/.103 | .429/.326 | .395/.356 | .463/.340 |  |
|  |  |  |  |  |  |
| CFI/RMSEA |  |  |  | .88/.058 | .88/.058 |
| AIC |  |  |  | 64614 | 64657 |
| BIC |  |  |  | 65171 | 64898 |
