# Supplementary material for: Unique developmental trajectories of risk behaviors in adolescence and associated outcomes in young adulthood
Source: PLoS One. 2019 Nov 13;14(11):e0225088. doi: 10.1371/journal.pone.0225088 (PMC6853606; doi:10.1371/journal.pone.0225088)
Supplement: S1 Fig — (DOCX) [file pone.0225088.s005.docx]

Supporting information

Figure S1. Path Diagram about decisions CFA analyses

Step 1

CFA each wave

Step 2

Check for MI with alcohol

Step 4

Check for sex differences

Step 3

Check for MI without alcohol

When partial MI:

-acceptable model fit

-poor factor loadings

When partial MI:

-Good model fit

-acceptable to low factor loadings

- Acceptable model fit

- poor factor loadings alcohol

-Good model fit

-Good factor loadings
