# Supplementary material for: Unique developmental trajectories of risk behaviors in adolescence and associated outcomes in young adulthood
Source: PLoS One. 2019 Nov 13;14(11):e0225088. doi: 10.1371/journal.pone.0225088 (PMC6853606; doi:10.1371/journal.pone.0225088)
Supplement: Attachment — Submitted filename: Review of U nique Developmental Trajectories of Risk Behaviors in Adolescence and Associated Outcomes in Young Adulthoo.docx [file pone.0225088.s006.docx]

Review of Unique Developmental Trajectories of Risk Behaviors in Adolescence and Associated Outcomes in Young Adulthood

This paper has examined 1) the construct of risk behavior at adolescent period 2) the development trajectory of risk behaviors, and 3) the association between development trajectory and outcomes at early adulthood. The purpose, data collection (measurement) are well specified. The statistical methods overall are appropriate. The study did not find there is a constant construct underlying the measured risk behaviors across age through CFA. For each risk behavior, the study revealed different developmental trajectory through latent growth modeling. In addition, through logistic regressions, the study found statistically significant relationship between interested outcomes and trajectories of risk behaviors but not the other.

The statistical methods applied are sufficient for the research purposes and the data properties, and the discussion of the missing data and its potential impact on investigated research questions is reasonable. Overall, the writing of the paper is well organized and clearly, though, there are a few places that needs clarification and improvement before publication.

*Method and Result presentation*

First, the authors indicated that CFA was performed to test the construct of risk behavior. However, it is not clear what specific model is applied to the data of multiple waves. It is assumed that first a configural invariance of single-factor model is specified to see if there is an underlying latent construct for smoking, cannabis use, and alcohol use, etc. If this model fit is not sufficient, it is not necessary to further test metric invariance or scalar invariance. It would be helpful if a path diagram is presented to show the structure of the model. Similarly, it would be helpful to present a summary table which display the factor loadings and test of significance.

Second, the labels for Tables need to have more details. For example, it is unclear what the numbers on Tables 1-4 represent, mean (SD). Also, although the authors have described in measurement section, it would be helpful if the table can standard alone with the necessary information. For Tables 5-8, please label reference group for the outcome variable, and OR (CI) on the head of the table wherever it is needed.

*Theoretical framework and Conclusion*

The author discussed the diverse definition of risk behavior in “Conceptualization of the risk behavior”. However, which conceptualization is adopted in this study is unclear. Is there other literature examining the structure of risk behavior construct using CFA? Second, “Risk” used in epidemiology is not the same as “risky decision making” are different. Only when a behavior/factor has potential negative impact on health outcome(s), that behavior is considered a risk behavior/factor. For example, “being sedentary” is a risk factor of obesity, but “being sedentary” is not “risk-taking”. Third, if a behavior is not considered risk in some age or group, the association between this behavior and later outcomes might have different interpretation.

The authors need to provide more information about study motivation and implication. For example, what is the motivation for studying development trajectory of risk behaviors. How will the knowledge of different development trajectories help with policy making and intervention.

Since age is an important factor in developmental trajectory, the author need to be more specific in review literature. For example, line 138-139, binge drinking at “what age” predicted lower school drop-out? It is also important to differentiate alcohol use and binge drinking, as some alcohol use is acceptable while binge drinking is not.
